# p97/VCP targets *Toxoplasma gondii* vacuoles for parasite restriction in interferon-stimulated human cells

**DOI:** 10.1128/msphere.00511-23

**Published:** 2023-11-17

**Authors:** Barbara Clough, Daniel Fisch, Todd H. Mize, Vesela Encheva, Ambrosius Snijders, Eva-Maria Frickel

**Affiliations:** 1 Institute for Microbiology and Infection, School of Biosciences, The University of Birmingham, Birmingham, United Kingdom; 2 Host-Toxoplasma Interaction Laboratory, The Francis Crick Institute, London, United Kingdom; 3 Advanced Mass Spectrometry Facility, School of Biosciences, The University of Birmingham, Birmingham, United Kingdom; 4 Proteomics Science Technology Platform, The Francis Crick Institute, London, United Kingdom; University at Buffalo, Buffalo, New York, USA

**Keywords:** *Toxoplasma gondii*, innate immunity, parasitophorous vacuole, ubiquitin, ANKRD13A, p97/VCP, UBXD1

## Abstract

Infection with the parasite *Toxoplasma gondii* leads to production of interferon gamma (IFNγ) that stimulates cells to upregulate defense proteins targeting the parasite for cell intrinsic elimination or growth restriction. Various host defense mechanisms operate at the parasitophorous vacuole (PV) in different human cell types leading to PV disruption, acidification, or membrane envelopment. Ubiquitin and p62 are players in all human host control mechanisms of *Toxoplasma*, but other unifying proteins have not been identified. Here, we show that p97/valosin-containing protein (VCP), as well as its associated proteins ANKRD13A and UBXD1 control *Toxoplasma* infection while recruited to the PV in IFNγ-stimulated endothelial cells. Convergent deposition of ANKRD13A, p97/VCP, and UBXD1 onto the same vacuole is dependent on vacuolar ubiquitination and observed within 2 h post-infection. ANKRD13A, p97/VCP, and UBXD1 all drive the acidification mechanism of the vacuole, which is the IFNγ-dependent control pathway of *Toxoplasma* in endothelial cells. We assessed p97/VCP in *Toxoplasma* control in various human cells and demonstrate that p97/VCP is a universal IFNγ-dependent host restriction factor targeting the *Toxoplasma* PV in epithelial (HeLa) and endothelial cells (human umbilical vein endothelial cells), fibroblasts (human foreskin fibroblast), and macrophages (THP1).

**IMPORTANCE:**

*Toxoplasma gondii* (Tg) is a ubiquitous parasitic pathogen, infecting about one-third of the global population. Tg is controlled in immunocompetent people by mechanisms that are not fully understood. Tg infection drives the production of the inflammatory cytokine interferon gamma (IFNγ), which upregulates intracellular anti-pathogen defense pathways. In this study, we describe host proteins p97/VCP, UBXD1, and ANKRD13A that control Tg at the parasitophorous vacuole (PV) in IFNγ-stimulated endothelial cells. p97/VCP is an ATPase that interacts with a network of cofactors and is active in a wide range of ubiquitin-dependent cellular processes. We demonstrate that PV ubiquitination is a pre-requisite for recruitment of these host defense proteins, and their deposition directs Tg PVs to acidification in endothelial cells. We show that p97/VCP universally targets PVs in human cells and restricts Tg in different human cell types. Overall, these findings reveal new players of intracellular host defense of a vacuolated pathogen.

## INTRODUCTION

*Toxoplasma gondii* (Tg) is a global human pathogen infecting one-third of the world’s population (1). Parasite infection leads to the production of the inflammatory cytokine interferon gamma (IFNγ) by natural killer and T cells. IFNγ acts on host cells to upregulate defense proteins that control the parasite and, in some cases, induces host cell death to eliminate the host replicative niche. Key to the success of Tg is its ability to replicate within many host species and infect most nucleated cells (2). The intracellular lifestyle of Tg is further protected as the parasite forms a vacuolar compartment from the host plasma membrane upon invasion, the parasitophorous vacuole, which it uses as a stronghold to mount counterattacks against its host (3).

IFNγ has long been shown to be the major player in the control of Tg with studies in mice revealing a dose-dependent effect of monoclonal antibodies to IFNγ on survival after Tg infection (4). The control of Tg tachyzoites in mice operates via mechanisms common to all murine cell types, involving two families of IFNγ-dependent GTPases, the immunity-related GTPases (IRGs), and guanylate-binding proteins (GBPs), with some Tg strains able to resist IRG-driven elimination. These GTPase families accumulate and co-ordinate a sequential, IRG-led attack on the PV resulting in vacuolar breakage and parasite death (5). In contrast to murine infection, study of Tg infection in human cells has revealed a complex interplay between host defense and Tg attack, with different cell types employing distinct mechanisms to combat the pathogen (6, 7). Many of the control mechanisms are dependent on the Tg strain- and IFNγ-dependent recruitment of ubiquitin to the PV within minutes of Tg invasion (8, 9). However, distinct from murine Tg infection, all Tg strains studied to date are eliminated in human cells.

Our study centers on understanding the cell-type-specific differences in Tg elimination in ubiquitin-marked vacuoles. Following ubiquitin targeting, host defense diverges to include recruitment of further host proteins to type II and III parasite vacuoles, including the autophagy-associated and ubiquitin-binding proteins p62 and NDP52. Host E3 ligases TRAF2 and TRAF6 have been implicated in bringing p62, NDP52, LC3B, and GABARAP to the Tg PV in IFNγ-stimulated primary human foreskin fibroblasts (HFFs), mediated by the parasite-secreted protein GRA15 (10). In the HeLa epithelial cell line, LC3 and GABARAPL2 also undergo ubiquitin-dependent accumulation at the PV leading to Tg growth restriction (9, 11). This contrasts with primary human umbilical vein endothelial cells (HUVEC) where vacuoles become LAMP1 positive and acidify leading to parasite death via a non-autophagic mechanism (8). In the latter two scenarios, the PV does not break, distinguishing these mechanisms strikingly from the IFNγ-driven murine host cell defense. In human cells, vacuole breakage has only been observed in immune cells such as the THP-1 macrophage cell line and primary blood monocyte-derived macrophages. Here, the vacuole is also ubiquitinated and subsequently decorated with GBP1 in type I and II strains of Tg, resulting in breakage of the PV membrane, culminating in cell death through an apoptotic pathway (12–14). More recently, in both A549 and HFF cells, the IFNγ-mediated control of *Toxoplasma* has been shown to be directed primarily by the IFNγ-inducible host E3-ligase RNF213, which promotes Lys63 and M1 ubiquitination at the PV leading to the recruitment of host defense proteins p62, NDP52, optineurin, and TAX1BP1 (15).

Host defense mechanisms also operate distal to the PV in human cell lines. Prior research has shown that IFNγ-dependent control of Tg in human fibroblasts could be attributed to tryptophan starvation of the tryptophan-auxotrophic Tg, through induction of host indoleamine 2,3-dioxygenase (IDO1) (16, 17). Classically viewed as PV-targeting host defense proteins, GBPs have been shown to also operate in PV-distal control of Tg. GBP1 is not observed at the vacuolar membrane in the lung epithelial cell line A549 but is instrumental in controlling parasite survival (18). GBP2 and GBP5 do not target the PV in human macrophages yet are able to restrict parasite growth in a GTPase-dependent fashion (19). This suggests a more complex interplay of host defense mechanisms working both distal to and at the vacuolar membrane.

The cell-specific molecular mechanisms of parasite control will become clearer as more host proteins are identified which co-operate with these ubiquitin-dependent host factors. Here, we identify p97/valosin-containing protein (VCP) as a protein that targets the Tg vacuole and operates in host defense in a range of human cell types.

## RESULTS

### ANKRD13A is a ubiquitin substrate in *Toxoplasma*-infected human cells

The PV of Tg type II Pru in epithelial and endothelial cells is ubiquitinated in a parasite strain- and IFNγ-dependent manner [Figure S1a and (8, 9)], with Lys63-linked ubiquitin being one of the dominant linkage types observed (8, 15). Ubiquitination is a key step in the recognition and tagging of parasite vacuoles for destruction, with the linkage pattern of ubiquitination determining the fate and function of the ubiquitinated substrates (20).

We took a proteomics approach using stable isotope labeling by amino acids in cell culture (SILAC) with mass spectrometry (MS) to identify new candidate ubiquitinated host proteins at the PV (Fig. S1b). Comparison of IFNγ-stimulated A549 epithelial cells, Tg-infected versus uninfected cells, was made using SILAC. Enrichment of ubiquitinated peptides by di-glycine immunoprecipitation was performed after tryptic digestion to enable quantitation of the changes in ubiquitination of host and parasite components on infection. This was followed by proteomic analysis using liquid chromatography coupled to tandem mass spectrometry (LC-MS/MS). A list of ubiquitinated host peptides specific for cells infected with Tg type II Pru was compiled with targets likely to be present at the PV identified from a distribution plot of target proteins (Fig. S1c; Table 1). We show that a host protein, ANKRD13A, is ubiquitinated on host cell infection by Tg type II Pru (Fig. S1c). ANKRD13A was of particular interest since this protein had been shown specifically to bind Lys63-linked ubiquitin chains and to be involved in membrane protein modulation and trafficking mechanisms (21, 22).

**TABLE 1:** The identification, by LC-MS/MS, of potential ubiquitin substrates listed with their respective peptide data^*a*^

Protein	PEP	Localization probability	C-term	Known site	Ratio H/L
ANKRD13A	8.36E−10	1			2.007
VAMP8	2.29E−3	0.87863	+	+	0.960
FYCO1	1.53E−3	1			1.549
LAMTOR1	6.82E−3	1		+	1.393
RHOB	5.04E−4	0.000504		+	3.659

^*a*^ Proteins containing ubiquitinated peptides from stable isotope labeling with amino acids in cell culture screen. Posterior error probability (PEP) gives the probability that observed peptide spectrum matches is incorrect.

### ANKRD13A, p97/VCP, and UBXD1 control *Toxoplasma* localized to the parasitophorous vacuole

**Fig 1:**
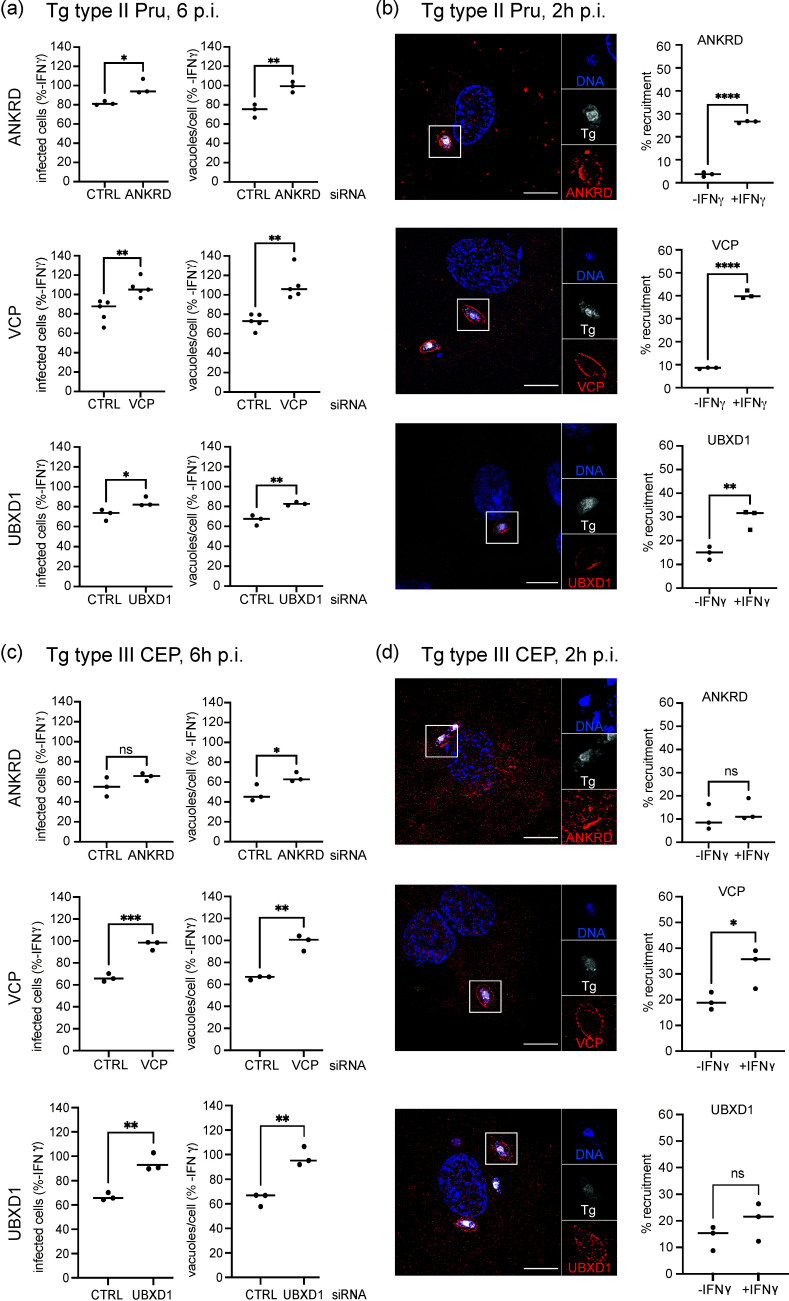
ANKRD13A, p97/VCP, and UBXD1 target Tg for IFNγ-driven restriction. (a) Target gene knockdown leads to an increased percentage of infected cells and more vacuoles/cell in IFNγ-stimulated HUVEC 6 h p.i. with Tg type II Pru compared to unstimulated cells. More than 1,000 cells counted. *n* = 3–5. (b) Representative structured illumination microscopy (SIM) image and quantification of target protein (red) recruitment to Tg type II Pru (white) vacuoles in HUVEC in dependence of IFNγ stimulation 2 h p.i. More than 200 vacuoles counted. Scale bar = 10 µm. (c) Target gene knockdown leads to an increased percentage of infected cells and more vacuoles/cell in IFNγ-stimulated HUVEC 6 h p.i. with Tg type III CEP compared to unstimulated cells. More than 1,000 cells counted. *n* = 3. (d) Representative SIM image and quantification of target protein (red) recruitment to Tg type III CEP (white) vacuoles in HUVEC in dependence of IFNγ stimulation 2 h p.i. Statistical significance: ns, not significant; *, *P* ≤ 0.05 **; *P* ≤ 0.01; ***, *P* ≤ 0.001; ****, *P* ≤ 0.0001. Scale bar = 10 µm.

ANKRD13A has been shown to interact with the AAA+ ATPase p97/VCP, which has many and diverse cellular roles with ubiquitinated substrate proteins being a common target for its functions (22, 23). Consequently, we wanted to address the fate of Tg in cells depleted of ANKRD13A and p97/VCP and the p97/VCP cofactor UBXD1. A combination of siRNAs for each of the proteins was transfected into HUVEC by nucleofection along with a non-targeting control siRNA. A marked reduction in all proteins was seen with the siRNA treatment by immunoblotting compared to the control siRNA (Fig. S2a). The percentage of infected cells and vacuole-to-cell ratio of Tg type II Pru were counted at 6 h post infection by automated high content imaging followed by host response to microbe analysis (HRMAn) (Fig. 1a) (24). A mean of 45% infected cells with Tg type II Pru was recorded in cells treated with siCTRL without IFNγ stimulation. Here, a significant increase in both the percentage of infected cells and vacuole-to-cell ratio was observed in cells where ANKRD13A, p97/VCP, or UBXD1 was knocked down (Fig. 1a). Furthermore, recruitment of all target proteins to Tg vacuoles was observed at 2 h p.i. for Tg type II Pru, with a significant increase in recruited vacuoles after stimulation of HUVEC with IFNγ (Fig. 1b). Similar observations were made for Tg type III CEP, where a mean of 34% infected cells with Tg type III CEP was recorded in cells treated with siCTRL without IFNγ stimulation. Although recruitment of ANKRD13A and UBXD1 was not significant, they both showed an increased trend in IFNγ-stimulated cells (Fig. 1c and d). To observe the kinetics of p97/VCP recruitment, live imaging was performed 2–3 h post-infection of EGFP-p97/VCP-transfected, IFNγ-stimulated HUVEC by Td-tomato stably transfected Tg type II Pru (Fig. S3a; Video S1). p97/VCP was observed to accumulate at the vacuole, and in one sequence, the parasite within the targeted PV loses fluorescence, indicating a loss of parasite viability and possible vacuolar acidification (Fig. S3ai; Video S1).

We conclude from these data that the host proteins ANKRD13A, VCP, and UBXD1 are most likely important in controlling the survival of Tg type II Pru and type III CEP and mediate their effect at the Tg vacuole.

### ANKRD13A, p97/VCP, and UBXD1 are ubiquitin-dependent host factors that target a PV subset

**Fig 2:**
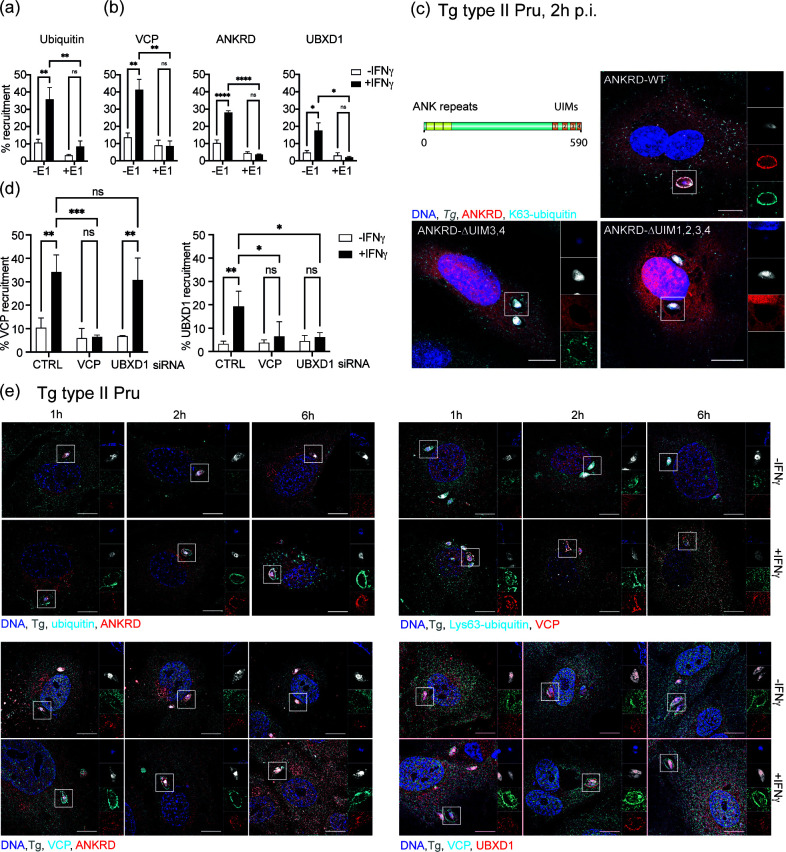
Ubiquitin-dependent recruitment of ANKRD13A, p97/VCP, and UBXD1 colocalizes to a subset of Tg vacuoles. (a) Total ubiquitin, (b) VCP, ANKRD13A, and UBXD1 are recruited to Tg type II Pru vacuoles in HUVEC 2 h p.i., in dependence of interferon gamma stimulation. Treatment of cells with E1 ubiquitination inhibitor PYR41 (+E1) significantly reduces the recruitment of these target proteins to Tg type II Pru vacuoles. More than 200 vacuoles counted, *n* = 3. (c) ANKRD13A is recruited to Tg type II Pru vacuoles through its ubiquitin UIM domains. Domain structure of ANKRD13A showing UIM domains with representative confocal images depicting over-expression of ANKRD13A UIM deletion mutants (red) and anti-Lys63-ubiquitin staining (cyan). Scale bar = 10 µm. (d) UBXD1 accumulation at Tg type II Pru vacuoles 2 h p.i. in IFNγ-stimulated HUVEC is dependent on p97/VCP recruitment, while VCP is able to target vacuoles in the absence of UBXD1. Recruitment of p97/VCP and UBXD1 to >200 Tg type II Pru vacuoles was determined at 2 h p.i. in cells knocked down for either p97/VCP or UBXD1. *n* = 3. (e) Host proteins target the same subset of Tg type II Pru vacuoles. Time course of denoted protein (red, cyan) co-recruitment to the Tg type II Pru (white) vacuole in HUVEC with or without IFNγ stimulation at 1, 2, and 6 h p.i. Representative structured illumination microscopy images are shown. Statistical significance: ns, not significant; *, *P* ≤ 0.05 **; *P* ≤ 0.01; ***, *P* ≤ 0.001; ****, *P* ≤ 0.0001 Scale bar = 10 µm.

Next, we wanted to determine whether ubiquitin was the driving factor for recruitment of ANKRD13A, VCP, and UBXD1, and whether these host proteins were directed to the same subset of PV. Recruitment of total ubiquitin, p97/VCP, ANKRD13A, and UBXD1 at 2 h was determined at the PV in cells pre-stimulated with IFNγ and then treated for 2 h with the E1 enzyme inhibitor, PYR41, prior to infection with Tg type II Pru. Without PYR41, IFNγ drives the deposition of ubiquitin onto the PV, and a reduction in ubiquitin recruitment in HUVEC stimulated with IFNγ confirmed the activity of the inhibitor (Fig. 2a). We observed significant decreases in recruitment of VCP, ANKRD13A, and UBXD1 in IFNγ-stimulated cells, revealing that ubiquitin was necessary for PV targeting by these host factors (Fig. 2b). ANKRD13A has been shown to bind Lys63-ubiquitinated substrates via ubiquitin-interaction motifs (UIMs) 3 and 4 located at its C-terminal end, with the interaction being regulated by its own monoubiquitylation (21). Using cells transfected with WT-ANKRD13A and mutants deleted in the C-terminal UIM domain, we found that mutants lacking UIMs 3 and 4 showed no observable localization to Lys63-ubiquitin-positive vacuoles (Fig. 2c). Furthermore, a lack of PV targeting was observed for the ANKRD13A UIM1-4 deletion mutant (Fig. 2c). Deletion of ANKRD13A’s UIM domains prevented observable accumulation of Lys63-ubiquitin at the PV (Fig. 2c).

As p97/VCP and UBXD1 are known to be cofactors for the endolysosomal damage response (ELDR) (25), we wanted to determine their respective hierarchy of recruitment to the PV. Knockdowns of p97/VCP and UBXD1 were made in HUVEC using siRNA, and recruitment of these proteins was scored at 2 h p.i., with specific protein recruitment to corresponding siRNA knockdown cells included as negative controls. While p97/VCP was still able to recruit to the PV in the absence of UBXD1, the converse was not observed (Fig. 2d). This showed that UBXD1 recruitment was only possible in the presence of p97/VCP. Since UBXD1 targeting to the PV requires both p97/VCP and ubiquitin, it implies that the UBXD1 association with p97/VCP occurs subsequent to p97/VCP localization to the PV. To assess whether these host proteins targeted the same PV, co-localization studies were performed at 1, 2, and 6 h post infection with Tg type II Pru (Fig. 2e). Deposition of host proteins onto the PV was absent or limited in the absence of IFNγ (Fig. 2e). This aligned with the slightly higher recruitment of these proteins in unstimulated cells observed in Fig. 1b. Ubiquitin and p97/VCP recruitment to type II PV in IFNγ-treated cells were apparent from 1 h p.i. with subsequent accumulation of ANKRD13A and UBXD1 from 2 h to ubiquitin- and p97/VCP-positive vacuoles (Fig. 2e). These data reveal that ubiquitin, ANKRD13A, p97/VCP, and UBXD1 are targeted to the same subset of vacuoles in IFNγ-stimulated cells with a hierarchy of recruitment. We show that ubiquitin drives recruitment to the vacuole during the first hour p.i. with p97/VCP accumulating on ubiquitinated PV from 1 h p.i. and subsequent targeting of p97/VCP-positive PVs by its cofactor UBXD1 and ANKRD13A after 2 h p.i.

### ANKRD13A, p97/VCP, and UBXD1 drive PV acidification with p97/VCP being a universal *Toxoplasma* PV-localized IFNγ-dependent human host protein

**Fig 3:**
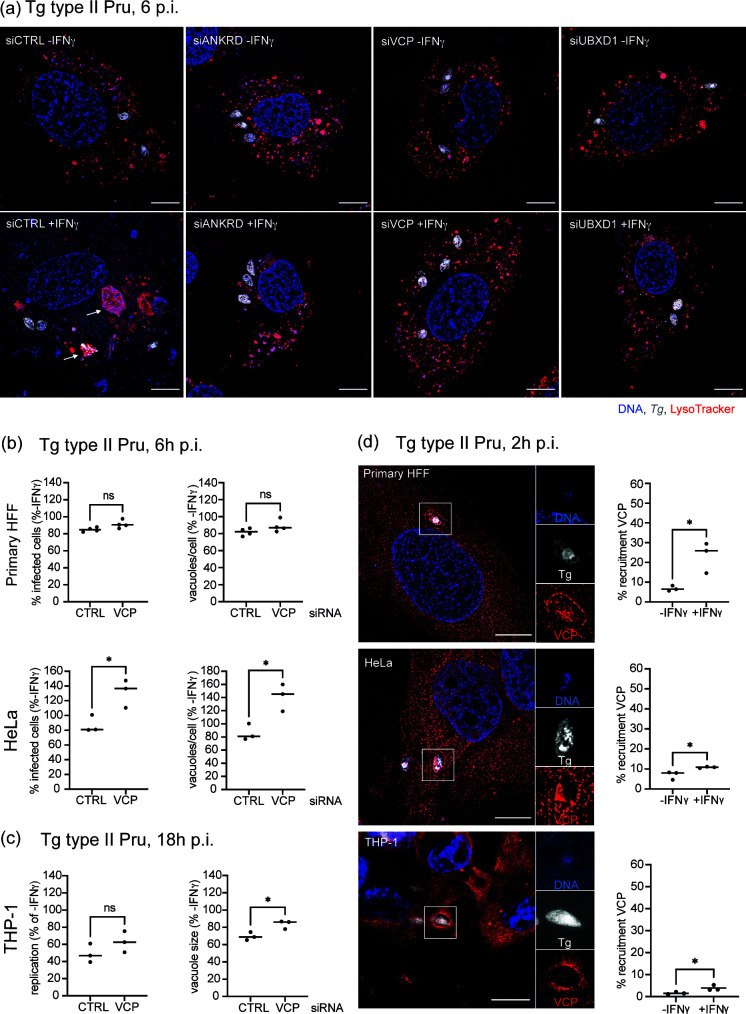
ANKRD13A, p97/VCP, and UBXD1 are host defense proteins driving PV acidification in HUVEC with p97/VCP impacting Tg in different cell types. (a) Host proteins ANKRD13A, VCP, and UBXD1 are involved in acidifying the Tg type II Pru vacuole in IFNγ-stimulated cells. Representative structured illumination microscopy (SIM) images of LysoTracker (red)-stained, Tg type II Pru (white) vacuoles 6 h p.i. in HUVEC knocked down with control (siCTRL) or target gene siRNA with and without IFNγ. White arrows indicate examples of acidified vacuoles. Scale bar = 10 µm. (b) p97/VCP knockdown did not lead to a significant increase in percentage of infected cells or vacuoles/cell in IFNγ-stimulated primary fibroblasts (HFF) infected with Tg type II Pru at 6 h p.i. compared to unstimulated cells. Depletion of p97/VCP led to an increased percentage of infected cells and more vacuoles/cell in IFNγ-stimulated human epithelial cells (HeLa) infected with Tg type II Pru at 6 h p.i. compared to unstimulated cells. More than 1,000 cells counted per condition, *n* = 3. (c) In the human macrophage (THP1) cell line, p97/VCP knockdown led to an increased vacuole size in IFNγ-stimulated cells with a trend toward increased replication. For THP1, readouts of percent replication and vacuole size were determined at 18 h p.i. since the level of cell death occurring in this cell type on infection interfered with analyses related to cell number. More than 1,000 cells counted per condition, *n* = 3. (d) IFNγ-dependent recruitment of VCP/p97 to Tg type II Pru vacuoles was observed at 2 h p.i. in primary HFF, HeLa, and THP-1 cells. Representative SIM images are shown with quantitation. Scale bar = 10 mm. More than 200 vacuoles were counted, *n* = 3.

Previously, we reported that Tg type II Pru is targeted for destruction in IFNγ-stimulated HUVEC by endolysosomal acidification, initiated by ubiquitination at the PV (8). To address whether the new host factors ANKRD13A, p97/VCP, and UBXD1 contributed to vacuolar acidification, we infected cells knocked down for each of the host proteins with Tg type II Pru for 6 h, adding LysoTracker to the cultures 1 h before fixation for microscopy. LysoTracker-stained vacuoles were clearly visible for parasite-infected control cells stimulated with IFNγ, whereas PV acidification was diminished in infected cells lacking the specified host proteins (Fig. 3a; Fig. S4). This supported the notion that ANKRD13A, p97/VCP, and UBXD1 are involved in clearance of Tg by acidification of the PV.

Since it has been shown that the mechanisms of parasite clearance vary in immune-stimulated human cells dependent upon cell type (26), we were curious to see whether the host factor p97/VCP was an important defense molecule in different cell types. p97/VCP has been previously implicated in intracellular pathogen survival (27, 28) and is further involved in diverse cellular processes involving membrane protein recycling and degradation in a range of cell types (23). Here, primary fibroblast (HFF) and endothelial (HeLa) cells knocked down for p97/VCP were infected with Tg type II Pru, and the impact on percentage infected cells and vacuoles/cell determined at 6 h p.i. using HRMAn analysis. In these experiments, mean control infection levels in the absence of IFNγ were 11% for HeLa, 20% for HFF, and 18% for THP1. While p97/VCP showed a significant effect on the IFNγ-dependent restriction of Tg in HeLa cells, there was little change observed in primary fibroblasts (Fig. 3b). This indicated that, at this time point, p97/VCP was important for the IFNγ-dependent control of Tg type II Pru in HeLa but not in primary HFFs. As THP-1 cells have been reported to undergo cell death upon IFNγ-induction and infection with Tg (12), we analyzed the infection readouts of percent replication and vacuole size at 18 h p.i.; parameters that do not depend upon total cell number. A significant increase in vacuole size was noted for immune-stimulated cells lacking p97/VCP, indicating that p97/VCP impacted the parasite’s ability to divide in macrophages (Fig. 3c). This was reflected in siCTRL treatment as a mean of 1.8 parasites per vacuole in unstimulated versus 1.3 in IFNγ-stimulated THP-1 cells, a 27% reduction, compared with siVCP knockdowns showing a mean of 1.6 parasites per vacuole in unstimulated cells and 1.5 in IFNγ-stimulated cells, a 6% reduction. We found that p97/VCP was recruited to Tg type II Pru vacuoles to different extents for primary fibroblasts, endothelial, and macrophage cell lines (Fig. 3d). IFNγ-dependent accumulation of p97/VCP increased significantly in all cell types with levels in primary fibroblasts higher than the other cell types.

**Fig 4:**
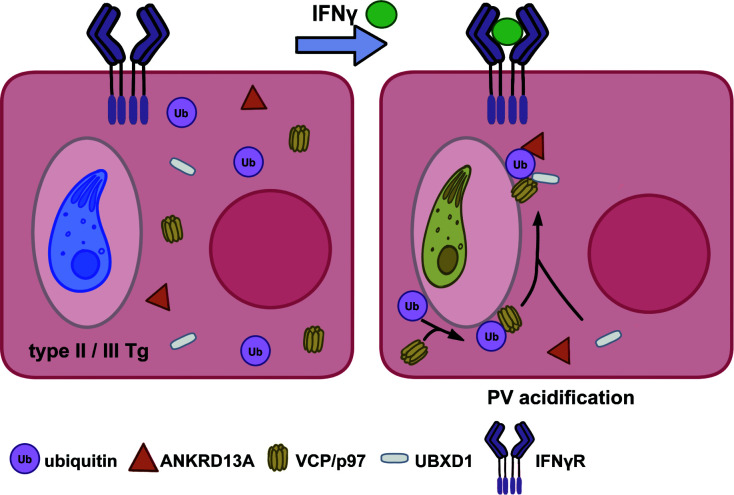
Model of p97/VCP interaction with *Toxoplasma gondii* vacuoles. Infection of interferon gamma-stimulated endothelial cells with the parasite *Toxoplasma gondii* culminates in the ubiquitin-led acidification of the parasite vacuole, observable within 6 h of infection. The AAA+ ATPase p97/VCP collaborates in this attack accompanied by its cofactor UBXD1 and a known interactor ANKRD13A. Along with ubiquitin, p97/VCP functions in vacuole-localized *Toxoplasma* control in diverse human cell types.

Collectively these data show that ANKRD13A, p97/VCP, and UBXD1 are host defense proteins that drive Tg type II Pru PVs to acidification in endothelial cells (Fig. 4). p97/VCP is universally recruited to different human cell types in dependence of IFNγ, with diverse effects on Tg restriction.

## DISCUSSION

Ubiquitination of the PV is a universal priming step in the IFNγ-driven control of *Toxoplasma* in human cells (3). We and others previously showed that ubiquitin-binding proteins p62 and NDP52 locate to the ubiquitinated PV and contribute to the elimination of Tg type II (8, 9). Here, we sought to examine pathways of Tg control that are seeded by PV ubiquitination. To this end, we employed MS to identify ANKRD13A as a novel ubiquitinated protein during Tg infection in IFNγ-stimulated epithelial cells. We explored this further by studying the ANKRD13A-associated proteins p97/VCP and UBXD1 at the PV and show that they act in defense against Tg infection in IFNγ-stimulated human cells. We demonstrate that these host defense proteins collaborated in the vacuolar acidification of Tg in endothelial cells (8). Furthermore, p97/VCP was recruited to the Tg PV in a diverse range of human cell types with a differential effect on Tg control. This implies that although universally recruited to the Tg PV in endothelial, epithelial, macrophage, and fibroblast cells, p97/VCP executes pathogen-defense functions in different human cell types possibly via divergent mechanisms.

Ankyrin repeat (AR) proteins, first isolated in mammalian erythrocytes, are involved in the targeting, mechanical stabilization, and orientation of membrane proteins to specialized compartments within the plasma membrane and endoplasmic reticulum (29). By extension, ANKRD13A may act as an adapter to recruit further proteins to the Tg PV. The ability of ANKRD13A to bind to Lys63-ubiquitinated substrates, as well as its role in endolysosomal trafficking, drew our attention to this protein as having potential relevance to the K63-ubiquitin-dependent endolysosomal destruction of Tg type II Pru that we have observed in primary endothelial cells (8). By ANKRD13A knockdown, we validated that the protein indeed controlled Tg in IFNγ-stimulated HUVEC, and using immunofluorescence microscopy, we verified its localization to the Tg type II Pru and type III CEP PV.

ANKRD13A is an AR protein with four ubiquitin interaction motifs that specifically bind Lys63-linked ubiquitin and has been postulated to act as a ubiquitin-dependent regulator of epidermal growth factor receptor (EGFR) internalization (21). The tuning of ANKRD13A activities by ubiquitin modulation has been further highlighted by its interaction with the E3 ligases RNF11 and ITCH (30). Here, the authors showed that ANKRD13A acts as a scaffold, allowing the transient formation of a complex between the E3 ligases ring finger protein 11 (RNF11) and Itchy E3 ubiquitin-protein ligase (ITCH) and EGFR, with the E3 ligases regulating the ubiquitination of ANKRD13A and thereby determining EGFR sorting for degradation or recycling. Tg has been reported to activate EGFR, albeit through an IFNγ-independent CD40 ligand-dependent mechanism, thus preventing autophagy-mediated killing of the parasite (31). It is plausible to speculate that in type I infections and infected cells not stimulated with IFNγ, ANKRD13A is not ubiquitinated and can bind and mediate EGFR internalization, thus enabling the parasite to evade host destruction via the acidification pathway. More recently, ANKRD13A has been implicated in the regulation of apoptotic cell death through its interaction with receptor interacting protein (RIP) 1 (32). Here, it binds ubiquitinated RIP1 preventing the recruitment of FAS-associated death domain protein (FADD) and caspase 8 to RIP1 preventing formation of cell death complex II. As mentioned above, ANKRD13A modulates ubiquitination of RNF11, an essential component of the A20 ubiquitin-editing protein complex, including ITCH and TAX1BP1 which can remove Lys63 ubiquitin from RIP1 (33). TAX1BP1 has also been shown to be recruited to Tg type II Pru vacuoles in A549 lung epithelial cells and in MIO-M1 Müller glial cells (15; B. Clough et al., unpublished data). Apoptotic cell death is employed in response to Tg infection of human macrophages as a mechanism of infection control, and it is conceivable that cellular interactions involving ANKRD13A may skew the response to Tg infection in different cell types (13).

In looking for ANKRD13A-associated proteins which may affect parasite survival, we noted an interaction with p97/VCP. ANKRD13A has been shown to facilitate the proteasomal degradation of caveolin-1 by forming a complex with p97/VCP, a hexameric AAA+ ATPase, and caveolin-1 (22). Its association with p97/VCP, a protein known for its ATP-dependent membrane segregase activity, led us to examine whether p97/VCP was also present at the PV and able to restrict *Toxoplasma*. We were able to verify that p97/VCP mediated IFNγ-dependent control on the Tg type II Pru and type III CEP and localized to the PV.

p97/VCP has many functions including the extraction of ubiquitinated proteins from membranes and serving as an interaction hub for >30 cofactors (34). Furthermore, p97/VCP is involved in modulating pathogen survival in both *Salmonella* and *Legionella*. In the former, a bacterial protein SptP regulates the activity of host p97/VCP by dephosphorylation, leading to increased intracellular replication of *Salmonella* (27). In the latter, p97/VCP was observed to promote bacterial replication by regulating the turnover of ubiquitinated proteins at the *Legionella*-containing vacuole (28). p97/VCP may similarly modify the protein architecture at the PV of Tg by removing ubiquitinated pathogen virulence or protective proteins. The function of p97/VCP is governed by its interaction with specific cofactors that mediate its subcellular localization as well as directing substrate binding, and p97/VCPs function in cellular processes and pathways (35). Although the mammalian p97/VCP cofactors number ~40, there are three major cofactors; p47, UFD1-NPL4, and UBXD1. Of these, UBXD1 has been linked to endolysosomal trafficking (36) and has been described as a cofactor for VCP in a range of co-operative roles, including the ELDR (25), mitophagy (37), endoplasmic reticulum-associated degradation (38), which links to our hypothesis of endolysosomal pathway for Tg PV acidification. Furthermore, we were unable to localize p47 to the PV in IFNγ-stimulated HUVEC, whereas UBXD1 was recruited to p97/VCP-positive vacuoles as shown in Fig. 2e.

To date, few universal host-defense operators have been observed at the PV in human cells. Notably, ubiquitin leads this vacuole-situated pathogen control, with p62 a prominent player in all cell types studied. The mechanistic role of p62 in different cell types, however, is unclear and may diverge between those cells where vacuole breakage and cell death predominate versus those restricting Tg within its PV.

Similarly, in this study, we show that p97/VCP is a host protein that not only targets PVs in different human cell types but additionally functions in Tg control. Since we observe varying levels of Tg survival with the knockdown of p97/VCP in different cells, this host protein may participate in Tg restriction through alternate pathways or at different stages of Tg and PV elimination. The variability in contribution of different host defense proteins to Tg control may in part be attributed to differences in IFNγR signal strength between different cell types and warrants further study. Additionally, it is noted that some host defense proteins restrict Tg through IFNγ-independent as well as IFNγ-dependent pathways in different cell types as observed for hGBP1 in A549 epithelial cells (18). Hence, multiple mechanisms of parasite restriction may well be operating at the same time.

Together, our findings suggest that a complex balance of ubiquitination and protein remodeling may be occurring at the PV, involving localized ANKRD13A, p97/VCP, and UBXD1 that could direct pathogen control toward different pathways dependent upon cell type.

## MATERIALS AND METHODS

List of reagents used in the study is provided in Table S1.

### Cell and parasite culture

Cultures of A549 lung alveolar epithelial cells (from Max Gutierrez) and human foreskin fibroblast cells (ATCC #SCRC-1041) for maintenance of *Toxoplasma* were grown in Gibco DMEM with GlutaMAX (Thermo Fisher Scientific #12077549) supplemented with 10% heat-inactivated FBS (Life Technologies) and cultured at 37°C in 5% CO_2_. Human umbilical vein endothelial cells (Promocell #C-12203) were cultured in Gibco Medium 199 (Thermo Fisher Scientific #11554426) supplemented with 20% heat-inactivated FBS, Heparin (Merck), and endothelial cell growth supplement (Merck #02-102). THP-1 monocyte cell line (TIB202, ATCC) was maintained in Gibco RPMI with GlutaMAX (Thermo Fisher Scientific #61870036) with 10% heat-inactivated FBS. THP1s were differentiated to macrophages with 50 ng/mL phorbol myristate acetate (PMA) (Merck #P1585) for 3 days and then rested for 2 days in PMA-free, complete medium.

*Toxoplasma gondii* stably expressing luciferase/EGFP or Td-tomato (Prugniaud type II and CEP type III) were maintained *in vitro* by serial passage on monolayers of HFF cells, cultured in Gibco DMEM with GlutaMAX (Thermo Fisher Scientific) supplemented with 1% FBS at 37°C in 5% CO_2_. All cell culture was performed without the addition of antibiotics. Cell lines and parasites were routinely tested for mycoplasma contamination by PCR test.

### Cell treatments

Cells were stimulated for 18–24 h in complete medium at 37°C with addition of 50–100U/mL human IFNγ (R&D Systems #285-IF-100).

The E1 inhibitor PYR41 (Tocris #2978) was added to cells prior to infection at a concentration of 50 µM and incubated at 37°C for 2 h. Before infecting cells with Tg, the cells were washed twice with warmed PBS so that the inhibitor did not impact ubiquitination within the parasite.

### *Toxoplasma gondii* infection

*Toxoplasma* were prepared from freshly 3× syringe-lysed (1 × 25G then 2 × 27G) HFF cultures. The parasite suspension was then briefly centrifuged at 50 × *g* for 3 min to pellet and remove cell debris before adding parasites to experimental cells at a multiplicity of infection (MOI) of 1–2:1. The cell cultures with added *Toxoplasma* were then centrifuged at 800 × *g* for 5 min to synchronize the infection, prior to culturing at 37°C, 5% CO_2_ for the required time.

### Stable isotope labeling with amino acids in cell culture

SILAC mass spectrometry was used to quantitate ubiquitination of host cell proteins in epithelial cells infected with Tg. A549 were cultured in SILAC heavy and light media containing 100 mg/L L-proline (DMEM containing “heavy” ^13^C-Lys and ^13^C-Arg, #K8R10 or “light” ^12^C-Lys and ^12^C-Arg, #K0R0 and supplemented with 10% dialyzed FBS), for six cell doublings to allow incorporation of ^13^C-amino acids (39). Both heavy and light A549 were transferred to corresponding SILAC media containing 0.5% dialyzed serum for 12 h before stimulating with 100 U/mL IFNγ for 18 h. The light A549 cells were then infected with syringe-lysed, type II *Toxoplasma* MOI = 4 for 2.5 h, after which infected cultures were washed with 3 × 10 mL PBS to remove free parasites. Heavy uninfected cultures and light infected cultures were resuspended using trypsin EDTA and mixed in a 1:1 ratio. The cell mixture was lysed with 9M urea in 20 mM HEPES pH8.0, supplemented with 1,000 U benzonase/10 mL urea, and sonicated on ice (3 mm probe, 50% amplitude, 3 × 15 s bursts). Chloroacetamide was added to 20 mM to inhibit deubiquitination enzymes. Protein concentrations were determined by Bradford Dye method (BioRad) with ~30 mg protein extracted in total. A final concentration of 10 mM dithiothreitol (DTT) (Sigma) was added to reduce the proteins and incubated at 37°C for 30 min. Next, the extracted proteins were alkylated using 20 mM chloroacetamide (Sigma) and incubated 30 min at RT in the dark. The alkylation reaction was quenched by addition of 10 mM DTT for 10 min at RT. Initial digestion with 15 µg Lys-C (Promega) was performed by incubation 2 h RT. An ~20 µg aliquot was taken to evaluate digestion by SDS-PAGE. The lysate was then diluted with 100 mM ammonium bicarbonate, 5% acetonitrile to a final urea concentration of <2M. Samples were then digested by addition of trypsin 1:100 enzyme to protein (wt/wt) and incubated 37°C overnight. The following day, the sample was digested two more times with trypsin (1:100, wt/wt) for 4 h each at 37°C.

### Enrichment of di-Gly peptides

Peptides containing the di-Gly remnant were enriched using K-ε-GlyGly affinity resin (Cell Signaling Technologies #5562), according to manufacturer’s protocol. Briefly, digests were reconstituted in 1.4 mL of immunoaffinity purification (IAP) buffer as supplied by the manufacturer. One aliquot (∼40 µL packed bead volume) was washed four times with PBS and mixed with the peptide sample. Incubation of sample and beads was performed with gentle rotation at 4°C for 2 h followed by a 30-s 2,000 × *g* spin to pellet the beads. The antibody beads were washed twice with ice-cold IAP buffer followed by three washes with ice-cold water. DiGly peptides were eluted from the beads with the addition of 50 µL of 0.15% TFA and allowed to stand at room temperature for 5 min. After a 30-s 2,000 × *g* spin, the supernatant was carefully removed and lyophilized for further LC-MS/MS analysis.

### LC-MS/MS

For MS analysis, peptides were resuspended in 0.1% TFA and loaded on 50 cm Easy Spray PepMap column (75 µm inner diameter, 2 µm particle size, ThermoFisher Scientific) equipped with an integrated electrospray emitter. Reverse phase chromatography was performed using the RSLC nano U3000 (Thermo Fisher Scientific) with a binary buffer system at a flow rate of 250 nL/min. Solvent A was 0.1% formic acid and 5% DMSO, and solvent B was 80% acetonitrile, 0.1% formic acid, and 5% DMSO. The diGly-enriched samples were run on a linear gradient of solvent B (2%–40%) in 90 min, total run time of 120 min including column conditioning. The nanoLC was coupled to a Q Exactive mass spectrometer using an EasySpray nano source (Thermo Fisher Scientific). The Q Exactive was operated in data-dependent mode acquiring higher-energy collision dissociation (HCD) MS/MS scans (*R* = 17,500) after an MS1 scan (*R* = 70,000) on the 10 most abundant ions using MS1 target of 1 × 10^6^ ions and MS2 target of 5 × 10^4^ ions. The maximum ion injection time utilized for MS2 scans was 120 ms, the HCD normalized collision energy was set at 28, the dynamic exclusion was set at 10 s, and the peptide match and isotope exclusion functions were enabled.

### MS data processing

Data processing was performed with MaxQuant software (version 1.3.0.5) as described previously (40). Parent ion and tandem mass spectra were searched against *Toxoplasma gondii* database. A list of 247 common laboratory contaminants provided by MaxQuant was also added to the database. For the search, the enzyme specificity was set to trypsin with maximum of three missed cleavages for the diGly data set and two missed cleavages for the rest of the data. The precursor mass tolerance was set to 20 ppm for the first search (used for mass re-calibration) and to 6 ppm for the main search. Carbamidomethylation of cysteines was specified as fixed modification; oxidized methionines and N-terminal protein acetylation were searched as variable modifications. Di-glycine-lysine was added to the list of variable modifications. The data sets were filtered on posterior error probability to achieve 1% false discovery rate on protein, peptide, and site level.

### siRNA knockdown

A combination of three siRNAs for ANKRD13A (ThermoFisher silencer select # s40093, s40094, s40095), two siRNAs for VCP (ThermoFisher silencer select # s14765, s14767) or three siRNAs for UBXD1 (ThermoFisher silencer select # s37291, s37292, s37293) and siRNA non-targeting control (AM4635) were used to transfect cells. HUVEC were nucleofected (AMAXA) with siRNAs (100 pmol) using HUVEC transfection reagent (VPB-1002, Lonza). Cells were used 48 h after transfection.

### Protein lysates, SDS-PAGE, and immunoblot

Cells were washed 3× in PBS 4°C, before lysis in 1% Triton-X100 in 25 mM Tris HCl, pH 7.4, 5 mM MgCl_2_, 150 mM NaCl plus 1× complete protease inhibitor cocktail III, without EDTA (Calbiochem). Lysates were put on a rotator at 4°C for 1 h, prior to centrifugation 18,000 rpm 4°C. Protein supernatant concentrations were determined by Bradford Dye Assay (BioRad) and 10 µg/lane separated by SDS-PAGE. Gels were dry blotted (iBlot, ThermoFisher) onto nitrocellulose membranes and probed with specified primary antibody diluted in PBS containing 5% BSA, 4°C overnight. Blots were washed 3 × 10 min in Tris-buffered saline plus 0.01% Tween 20 (TBS/Tween) before probing with relevant secondary antibody diluted in TBS/Tween. After washing 3 × 10 min in TBS/Tween, blots were rinsed in TBS and developed with Immobilon Western Chemiluminescence HRP substrate (Millipore) and imaged on Imager A680 (Amersham).

### High content imaging and analysis

HUVEC were plated at 10 K cells/well in 96 well plates (Imaging plates; Falcon black-walled #353219) and were later IFNγ stimulated after cells had attached. After 18 h, cells were infected with type II *Toxoplasma-*GFP at MOI = 1–2 for 6 h. Plates were washed in PBS to remove uninvaded parasites and fixed in 4% PFA 15 min. The cells were permeabilized with PB (0.2% BSA, 0.02% Saponin in PBS) for 30 min before staining with Hoechst 33342 5 ug/mL and Cell Mask Red (ThermoFisher #H32712). After washing twice with PBS, the stained plates were imaged on Zeiss Cell Discoverer 7 using 20× objective and 0.5× magnification. Images were analyzed using Zen Blue software, and infection analysis performed by host response to microbe analysis pipeline to obtain values for percentage infection and vacuole/cell ratio. For recruitment analysis, cells were plated and infected for 2 h before fixation as above and were then permeabilized and stained with antibody, according to the immunofluorescence protocol below. Recruitment was counted manually (>200 vacuoles per condition) since the HRMAn pipeline had not been trained for these cells and stains.

### Immunofluorescence microscopy

HUVEC were plated on coverslips (12 mm, ♯1.5, ThermoFisher) in a 24-well plate and cultured, IFNγ stimulated, and infected with *Toxoplasma* as described above. All following steps were carried out at RT. The cells were washed with PBS and fixed with 3% paraformaldehyde in PBS for 15–20 min. The fix was aspirated, and Perm-Quench solution (50 mM NH_4_Cl, 0.2% (wt/vol) saponin, Sigma 47036 in PBS) added as a wash and replaced with fresh Perm-Quench and incubated for 10–15 min. The Perm-Quench solution was replaced with PGAS (0.2% [wt/vol] fish gelatin, Sigma G-7765, 0.02% [wt/vol] saponin, 0.02% [wt/vol] NaN_3_ in PBS). Cells were incubated for at least 5 min, and fixed cells could be stored in PGAS at 4°C for several days. Antibody incubations were carried out in a humid box, inverting coverslips onto 50 µL drops of primary antibody, diluted in PGAS, and incubating for 1 h at RT (except anti-LC3B: overnight incubation 1:100 at RT). Coverslips were washed in 3 × 1 mL volumes of PGAS before incubating for a further 1 h with second antibody, diluted in PGAS, at RT in the dark. Washes of 3 × 1 mL PGAS and 2 × 1 mL PBS followed by 1 mL PBS containing 1 µg/mL Hoechst 33342 (Life Technologies) and finally two washes in dH_2_O prior to mounting on glass slides with Mowiol 4–88 (Polysciences Inc.). Mounting medium was allowed to harden overnight. Slides were viewed on an SP5-invert Confocal microscope using ×100 objective and analyzed using LAS-AF software or by structured illumination microscopy (SIM) on Zeiss Elyra 7 using ×63 objective and Zen Black imaging software. Composite images were assembled using FiJi software.

### Live microscopy

HUVEC were transfected by nucleofection using Neon according to manufacturer’s protocol with pEGFP-p97, which was a gift from Hemmo Meyer (Addgene plasmid #85670; https://www.addgene.org/85670). After 24 h, cells were stimulated with IFNγ and infected the following day with Tg type II Pru-td-Tomato. Imaging was carried out in an environmentally controlled chamber, 37°C, 5% CO_2_ using a Nikon LTTL widefield microscope. Data were collected and analyzed using Micro-Manager and FiJi.

### Data handling, statistical measurements, and evaluation

Numerical data were plotted using Graph Pad Prism and presented as mean ± SEM. Significance of results was determined by two-way ANOVA or two-tailed Student *t*-test.

## Data Availability

The mass spectrometry proteomics data have been deposited to the ProteomeXchange Consortium via the PRIDE (41) partner repository with the data set identifier PXD042937. All other data are available from the corresponding author on request.

## SUPPLEMENTAL MATERIAL

Fig. S1 msphere.00511-23-s0001.pdf Mass spectrometry identifies ANKRD13A as a ubiquitinated protein during *Toxoplasma* (Tg) infection.

Fig. S2 msphere.00511-23-s0002.pdf Control of gene knockdowns using siRNA interference.

Fig. S3 msphere.00511-23-s0003.pdf Recruitment kinetics of p97/VCP to *Toxoplasma* (Tg) type II Pru vacuoles.

Fig. S4 msphere.00511-23-s0004.pdf Vacuolar acidification of Tg type II Pru at 6 h p.i.

Supplemental Text msphere.00511-23-s0005.docx Supplemental figure legends, Table S1, and Video S1 legend.

Video S1 msphere.00511-23-s0006.mp4 Dynamic recruitment of p97/VCP to *Toxoplasma* (Tg) type II Pru vacuoles.

ASM does not own the copyrights to Supplemental Material that may be linked to, or accessed through, an article. The authors have granted ASM a non-exclusive, world-wide license to publish the Supplemental Material files. Please contact the corresponding author directly for reuse.
