# Supplementary figures and images for: p97/VCP targets Toxoplasma gondii vacuoles for parasite restriction in interferon-stimulated human cells
Source: mSphere. 2023 Nov 17;8(6):e00511-23. doi: 10.1128/msphere.00511-23 (PMC10732073; doi:10.1128/msphere.00511-23)

FIGURE S1

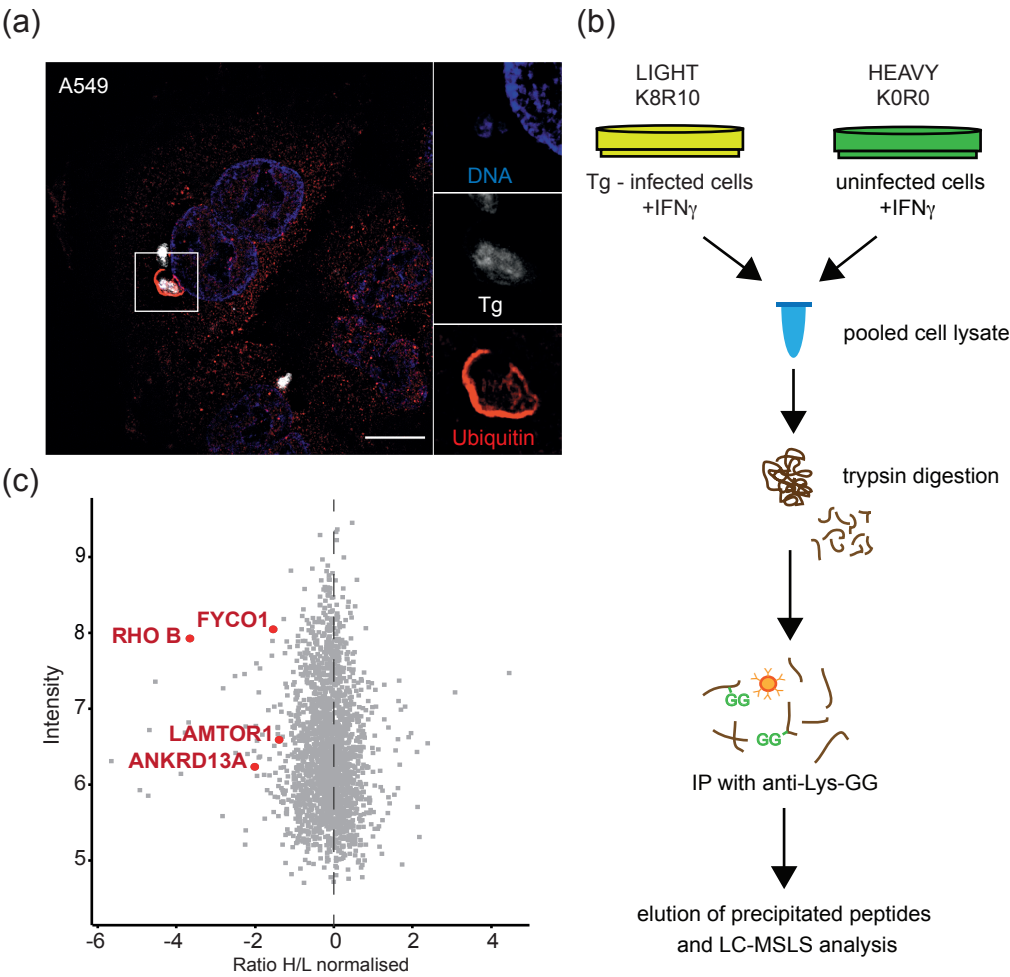

Supplement: Fig. S1 — Mass spectrometry identifies ANKRD13A as a ubiquitinated protein during Toxoplasma (Tg) infection. [file msphere.00511-23-s0001.pdf]

FIGURE S2

(a) HUVEC

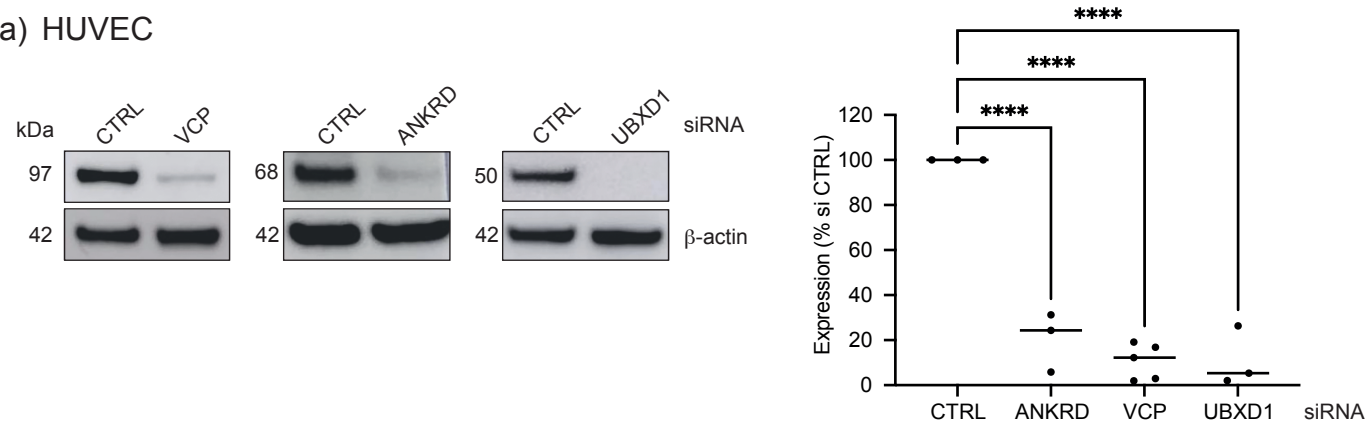

(b) HFF

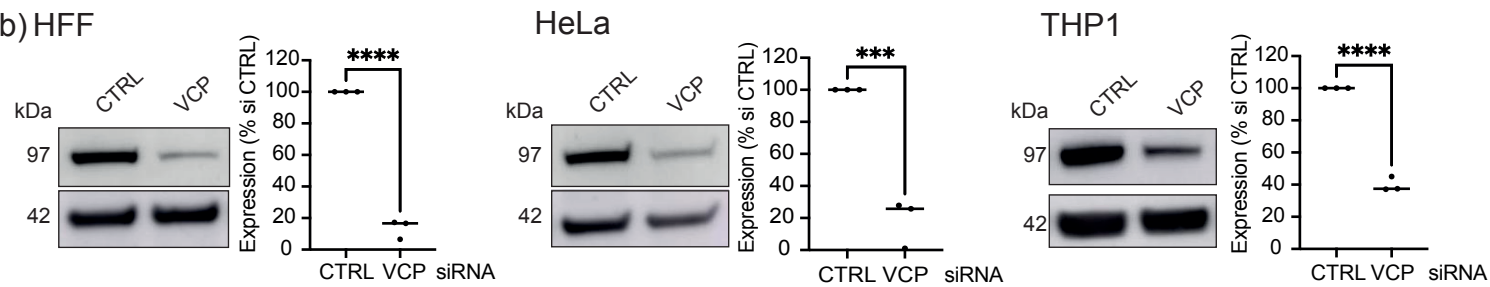

HeLa

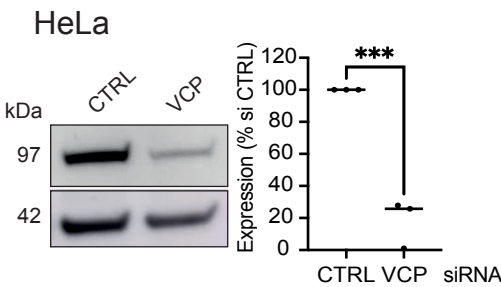

THP1

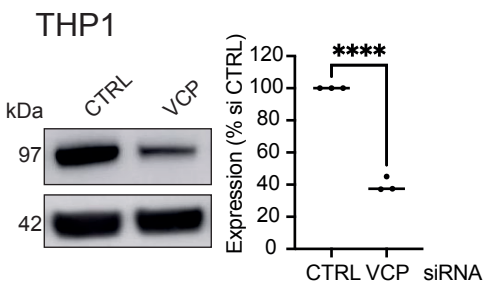

Supplement: Fig. S2 — Control of gene knockdowns using siRNA interference. [file msphere.00511-23-s0002.pdf]

FIGURE S3

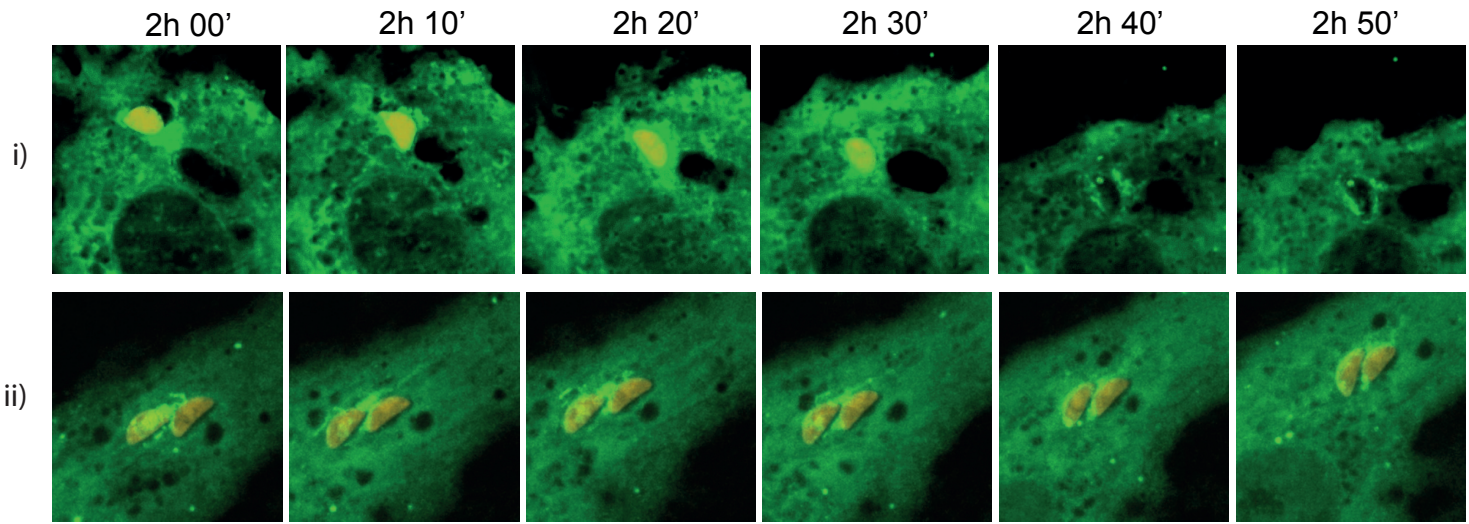

EGFP-p97/VCP 2-3h p.i. Tg type II Pru +IFN $\gamma$

Supplement: Fig. S3 — Recruitment kinetics of p97/VCP to Toxoplasma (Tg) type II Pru vacuoles. [file msphere.00511-23-s0003.pdf]

FIGURE S4

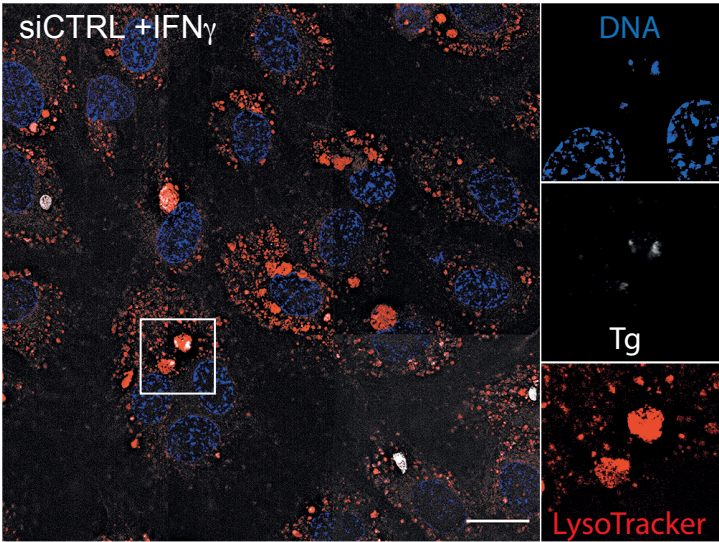

Supplement: Fig. S4 — Vacuolar acidification of Tg type II Pru at 6 h p.i. [file msphere.00511-23-s0004.pdf]
