# Supplementary material for: p97/VCP targets Toxoplasma gondii vacuoles for parasite restriction in interferon-stimulated human cells
Source: mSphere. 2023 Nov 17;8(6):e00511-23. doi: 10.1128/msphere.00511-23 (PMC10732073; doi:10.1128/msphere.00511-23)
Supplement: Supplemental Text — Supplemental figure legends, Table S1, and Video S1 legend. [file msphere.00511-23-s0005.docx]

**Supplementary Figure Legends**

**FIGURE S1. Mass Spectrometry identifies ANKRD13A as a ubiquitinated protein during *Toxoplasma* (Tg) infection.**

1. Representative structured illumination microscopy (SIM) image showing that the vacuoles of Tg type II Pru (white) in epithelial (A549) cells is ubiquitinated (red) in interferon gamma (IFNγ)-stimulated cells, nuclei (blue). Scale bar = 10μm.
2. A scheme for the stable isotope labelling by amino acids in culture (SILAC) workflow to identify substrates of ubiquitin is represented.
3. Intensity plot showing heavy/light ratio, for ubiquitinated peptides, with proteins of interest highlighted in red.

**FIGURE S2. Control of gene knockdowns using siRNA interference.**

1. Immunoblots and quantitation showing protein levels in interferon gamma (IFNγ)-primed HUVEC treated with control or target-specific siRNA. Representative blots of 3-5.
2. Immunoblots and quantification of protein expression after control and siRNA knockdown of VCP/p97 in HFF, HeLa and THP1 cells. Representative blots of 3.

**FIGURE S3. Recruitment kinetics of p97/VCP to *Toxoplasma* (Tg) type II Pru vacuoles.**

Live microscopy showing dynamic recruitment of EGFP-p97/VCP (green) to Tg type II Pru vacuoles (red) from 2h-2h50’ p.i. in interferon gamma (IFNγ)-stimulated HUVEC. Two cells i) and ii) are shown.

**FIGURE S4. Vacuolar acidification of Tg type II Pru at 6h p.i.**

Representative stitched structured illumination microscopy (SIM) image showing LysoTracker (red)-stained acidification of Tg type II Pru (white) vacuoles 6h p.i. in IFNγ-stimulated HUVEC. Scale bar = 30μm.

**Supplementary Video Legend**

**VIDEO S1. Dynamic recruitment of p97/VCP to *Toxoplasma* (Tg) type II Pru**

**vacuoles.**

Live microscopy showing dynamic recruitment of EGFP-p97/VCP (green) to Tg type II Pru vacuoles (red) from 2h-2h50’ p.i. in interferon gamma (IFNγ)-stimulated HUVEC.

**Supplementary Table S1:** List of reagents used in this study

| **Reagent or resource** | **Supplier** | **Catalogue number** |
| --- | --- | --- |
|  |  |  |
| **Antibodies** |  |  |
|  |  |  |
| Lys-ε-Gly-Gly (diGly) affinity resin | Cell Signalling Technologies | #5562 |
| ANKRD13A, rabbit polyclonal | Proteintech | #23998-1-AP |
| Ubiquitin Lys-63 specific, clone Apu3, rabbit monoclonal | Merck Millipore | #05-1308 |
| Ubiquitin, clone FK2, mouse monoclonal | Merck Millipore | #04-263 |
| p97/VCP, mouse monoclonal | Abcam | #ab11433 |
| p97/VCP, mouse monoclonal | ThermoFisher Scientific | #MA3-004 |
| UBXD1, rabbit polyclonal | Abcam | #ab221167 |
| UBXD1, rabbit polyclonal | Proteintech | #14704-1-AP |
| goat anti-rabbit IgG Alexa Fluor 568 | ThermoFisher Scientific | #A11036 |
| chicken anti-rabbit IgG Alexa Fluor647 | ThermoFisher Scientific | #A31573 |
| goat anti-mouse IgG Alexa Fluor568 | ThermoFisher Scientific | #A11004 |
| donkey anti-mouse IgG Alexa Fluor647 | ThermoFisher Scientific | #A21463 |
| anti-rabbit IgG HRP conjugated | Cell Signalling Technologies | #7074P2 |
| rabbit anti-mouse IgG (H+L) HRP conjugated | Merck | #AP160P |
|  |  |  |
| **Reagents** |  |  |
|  |  |  |
| Hoechst 33342 | ThermoFisher Scientific | #3570 |
| LysoTracker red DND-99 | ThermoFisher Scientific | #L7528 |
| Phosphostop | Roche | #04 906 837001 |
| Protease Inhibitor Cocktail III | Merck Millipore | #5539134-1SET |
| Dulbecco’s Phosphate Buffered Saline | Sigma-Aldrich | #D8537 |
| Immobilon Western Chemiluminescence HRP Substrate | Merck Millipore | #WBKLS0500 |
| Novex Sharp pre-stained protein standards | Thermo Fisher Scientific | #5800 |
| Protein loading dye 3x | New England Biolabs | #B7703S |
| Foetal bovine serum (FBS) | Thermo Fisher Scientific | #A5256701 |
| PYR41 (E1 inhibitor, Tocris) | BioTechne | #2978 |
| Gibco^TM^ DMEM with GlutaMAX | Thermo Fisher Scientific | #12077549 |
| Gibco^TM^ Medium 199 | Thermo Fisher Scientific | #31150022 |
| Gibco^TM^ RPMI with GlutaMAX | Thermo Fisher Scientific | #61870036 |
| Heparin | Merck | #H3149 |
| ECGS | Merck | #02-102 |
| PMA | Merck | #P1585 |
| Interferon gamma (recombinant) | R&D Systems | #285-IF-100 |
| Trypsin EDTA 0.5% (10x) | Thermo Fisher Scientific | #15400054 |
| Bradford Dye reagent | BioRad | #500-0006 |
| Dithiothreitol (DTT) | Sigma | #D9779 |
| LysC | Promega | #V1671 |
| Dimethyl sulphoxide (DMSO) | Merck | #D2650 |
